# Mycorrhizal fungi control phosphorus value in trade symbiosis with host roots when exposed to abrupt ‘crashes’ and ‘booms’ of resource availability

**DOI:** 10.1111/nph.17055

**Published:** 2020-11-29

**Authors:** Anouk van't Padje, Gijsbert D. A. Werner, E. Toby Kiers

**Affiliations:** ^1^ Laboratory of Genetics Wageningen University & Research Droevendaalsesteeg 1 Wageningen 6708 PB the Netherlands; ^2^ Department of Ecological Sciences Faculty of Earth and Life Sciences Vrije Universiteit de Boelelaan 1085 Amsterdam 1081 HV the Netherlands; ^3^ Department of Zoology University of Oxford Oxford OX1 3PS UK; ^4^ Netherlands Scientific Council for Government Policy Buitenhof 34 The Hague 2513 AH the Netherlands

**Keywords:** arbuscular mycorrhizal fungi, biological markets, quantum dots, symbiosis, trading strategies

## Abstract

- Biological market theory provides a conceptual framework to analyse trade strategies in symbiotic partnerships. A key prediction of biological market theory is that individuals can influence resource value – meaning the amount a partner is willing to pay for it – by mediating where and when it is traded. The arbuscular mycorrhizal symbiosis, characterised by roots and fungi trading phosphorus and carbon, shows many features of a biological market. However, it is unknown if or how fungi can control phosphorus value when exposed to abrupt changes in their trade environment.
- We mimicked an economic ‘crash’, manually severing part of the fungal network (*Rhizophagus irregularis*) to restrict resource access, and an economic ‘boom’ through phosphorus additions. We quantified trading strategies over a 3‐wk period using a recently developed technique that allowed us to tag rock phosphate with fluorescing quantum dots of three different colours.
- We found that the fungus: compensated for resource loss in the ‘crash’ treatment by transferring phosphorus from alternative pools closer to the host root (*Daucus carota*); and stored the surplus nutrients in the ‘boom’ treatment until root demand increased.
- By mediating from where, when and how much phosphorus was transferred to the host, the fungus successfully controlled resource value.

## Introduction

Underground, arbuscular mycorrhizal fungi and plant roots form a trade partnership to exchange resources. The fungus depends on the host plant for carbon (C) (sugars and fats) to fuel the development of a hyphal network foraging for nutrients in the soil (Jiang *et al*., 2017; Luginbuehl *et al*., 2017). In return, the fungus delivers phosphorus (P) and nitrogen (N), and other essential elements to the host root (Luginbuehl & Oldroyd, 2017; Field *et al*., 2019). These nutrients are often physically and chemically less accessible to the host, but can be taken up by the fungus and actively transported through the mycelium (Timonen *et al*., 2001). With evolutionary origins dating back roughly 450 Myr, the mycorrhizal symbiosis, emerging from the Mucoromycotina and/or Glomeromycota (Field *et al*., 2015; Hoysted *et al*., 2018; Strullu‐Derrien *et al*., 2018), is responsible for mediating nutrient cycles (Johnson *et al*., 2016), and transferring *c*. 5 billion tons of C yr^–1^ from plants to belowground ecosystems (Redecker *et al*., 2000; Bago *et al*., 2000).

Unlike many symbioses, which involve one‐to‐one or one‐to‐many interactions, the mycorrhizal symbiosis is characterised by multiple partners transferring nutrients to multiple partners simultaneously (Field & Pressel, 2018). A single plant host is colonised by multiple fungal species and a single fungal mycelium network can colonise multiple plant hosts (Selosse *et al*., 2006; Montesinos‐Navarro *et al*., 2012). These interactions mean that there is potential variation in resource transfer depending on environmental context, for example the number of partners (Hart *et al*., 2013; Perez‐Lamarque *et al*., 2020), the quality of those partners (Lekberg *et al*., 2010; Kiers *et al*., 2011; Argüello *et al*., 2016) and the local resource availability (Johnson *et al*., 2015; Ji & Bever, 2016; Whiteside *et al*., 2019).

Variation in resource transfer is a defining feature of so‐called ‘biological markets’ in which partners compete to provide resources, and those offering the best rate of exchange are favoured (Tasoff *et al*., 2015; Hammerstein & Noë, 2016; Noë & Kiers, 2018). Biological market theory argues that exchanges of resources and services among organisms can be analysed in market terms, with individuals making strategic trading investments depending on the context of the exchange (Werner *et al*., 2014). However, it is unknown how applicable the theory is across diverse systems, as it requires that individuals are able to compare and discriminate among competing traders. Past work has demonstrated that some plants are able to discriminate among competing fungal strains, and allocate more C to the higher quality fungi (Kiers *et al*., 2011; Grman, 2012; Ji & Bever, 2016; Werner *et al*., 2018). It has likewise been shown that fungal partners allocate more P to plant partners providing more C (Lekberg *et al*., 2010; Fellbaum *et al*., 2012, 2014; Konvalinková *et al*., 2015). Empirical work on partner discrimination is similarly supported by theoretical work showing that the act of individuals dividing resources among trading partners, in direct relationship to the relative amount of resources they receive, is the predicted outcome of natural selection (Wyatt *et al*., 2014).

Although the principles of biological market theory are generally well supported in mycorrhizal studies, there are also well documented examples of resource exchange between plants and fungi that do not follow market theory predictions (Olsson *et al*., 2010; Walder *et al*., 2012; Walder & van der Heijden, 2015; Field & Pressel, 2018; Charters *et al*., 2020). Some of these examples, including elegant work on mycoheterotrophic plant species (Bidartondo, 2005; Courty *et al*., 2011; Gomes *et al*., 2019; Perez‐Lamarque *et al*., 2020), are clear illustrations of how reciprocal exchange patterns can be violated depending on context. While neither the disappearance of cheating nor perfect partner choice is predicted by biological market theory (Kiers *et al*., 2016; Noë & Kiers, 2018), many open questions remain on the mechanisms that underlie recipiorocal exchange patterns. One of these questions is how partners respond to abrupt changes in local resource abundance and if/how they adapt their trade patterns to these conditions.

A key prediction of biological market theory is that partners can control or influence the ‘exchange rate’ (amount received : given) as resource availability becomes higher or lower (Noë & Hammerstein, 1994, 1995; Hammerstein & Noë, 2016). For mycorrhizal symbiosis, the exchange rate is defined as the units of C that a fungus receives from a partner plant per unit P provided by the fungus (Noë & Kiers, 2018). Because carbon allocation to the fungal symbiont is costly (*c*. up to 20% of carbon, Douds *et al*., 1988; Jakobsen & Rosendahl, 1990), the host is expected to respond to changes in fungal phosphorus transfer by modifying how much carbon it allocates to the fungus (i.e. reciprocal rewards, Kiers *et al*., 2011). From the fungal vantage point, the value of phosphorus increases if the host is willing to pay more for it (Whiteside *et al*., 2019). Quantitatively, this value is defined by a higher C : P exchange rate for the fungus. However, measuring how exchange rates respond to changing resource availability has been challenging because of difficulties in precisely manipulating resource availability in mycorrhizal systems, and quantifying resource transfer over time.

**Fig. 1:**
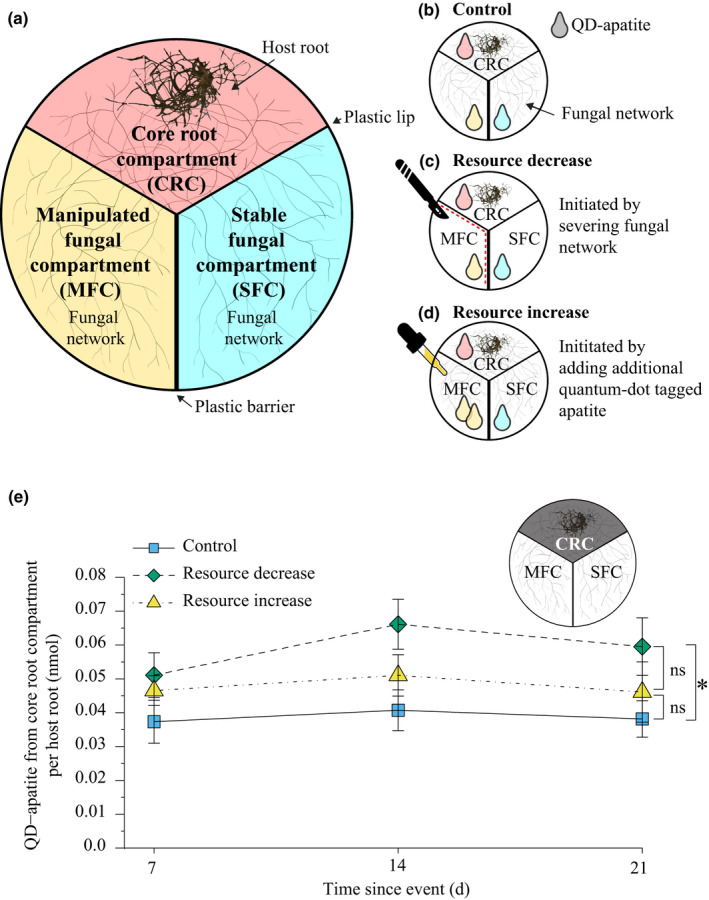
Experimental design and quantum‐dot (QD) apatite from core root compartment (CRC) per host root (*Daucus carota*). (a) Petri plates were divided into three compartments separated by a plastic lip (crossable by fungus) between the core root and the two fungal compartments (manipulated fungal compartment (MFC) and stable fungal compartment (SFC)) and a plastic barrier between the MFC and SFC (not crossable by fungus). The host root was grown in the CRC, which was filled with standard Modified Strullu–Romand (MSR) medium. The two fungal compartments, the MFC and the SFC, were filled with adjusted MSR medium with no added phosphorus (P). (b) In each treatment, P was supplied as QD‐apatite. Red QD‐apatite was added to the CRC, yellow QD‐apatite to the MFC, and cyan QD‐apatite to the SFC. (c) In the resource decrease treatment, the hyphae (*Rhizophagus irregularis*) were severed crossing the plastic lip into the MFC to restrict P from that compartment. (d) In the resource increase treatment, additional QD‐apatite was added to the MFC to double the P availability. (e) The amount of QD‐apatite from the CRC in the host roots was significantly influenced by treatment (two‐way ANOVA for treatment: *F*_2,132_ = 6.955, *P* = 0.001; time: *F*_2,132_ = 1.035, *P* = 0.358; interaction: *F*_4,132_ = 0.248, *P* = 0.911). Higher amounts of QD‐apatite from the CRC were found in the resource decrease treatment (green diamonds), than in the control (blue squares) or resource increase treatment (yellow triangles). The amount of QD‐apatite was not significantly different between the control and resource increase treatment. *n*_control,7_ = 14, *n*_control,14_ = 14, *n*_control,21_ = 21, *n*_decrease,7_ = 16, *n*_decrease,14_ = 18, *n*_decrease,21_ = 13, *n*_increase,7_ = 16, *n*_increase,14_ = 17, *n*_increase,21_ = 14; mean ± SE. Significant differences between the treatments are indicated by an asterisk; ns, nonsignificant differences.

We recently developed a fluorescing tagged P that can help resolve some of these constraints (Whiteside *et al*., 2019; van’t Padje *et al*., 2020). Specifically, we tagged apatite, a form of rock phosphate, with nanoparticles called quantum dots (QDs) that fluoresce bright and pure colours when excited with UV light. We tagged apatite with a class of QDs that fluoresce in different colours depending on chemical composition of the QD core (Fig. 1a), with each colour having the same size and weight (Jang *et al*., 2003; Bailey & Nie, 2003; Whiteside *et al*., 2019). The outer layer of carboxyl polymers protects the organisms from the toxicity of the heavy metal core, and allowed us to conjugate the QDs to P. In this study, we use our QD‐apatite technique to synthesise three colours of fluorescent QD‐apatite. We added a single colour of QD‐apatite to each of three compartments. The root compartment contained the *in vitro* colonised mycorrhizal root. The other two fungus‐only compartments contained the symbiotic fungal network (i.e. filamentous mycelium of arbuscular mycorrhizal fungi) (Fig. 1).

Our aim was to determine how the P transfer strategies of the fungus changed in response to sudden changes in resource availability. In biological market language, abrupt and extreme changes in resource availability are analogous to market ‘crashes’ and ‘booms’ because resource access to one partner is directly enhanced or diminished. In nature, this can happen for example when fungal networks experience regional changes in edaphic conditions (e.g. Stevens *et al*., 2020) such an influx of nutrients or when they are cut off from existing nutrient sources.

Specifically, it is unknown if the exchange rate (C : P ratio) will change when the fungus is exposed to a sudden change in resource availability. The fungus, for example, could modify when, from where or how much nutrients it transfers to the host. This has the potential to change the value, with the fungus mediating the C : P exchange rate. Therefore, we monitored the transfer of QD‐apatite from the fungus to the host under three different resource treatments. To mimic a market ‘crash’, we manually severed the fungal network growing in one of the compartments to abruptly restrict QD‐apatite access (Fig. 1c). Likewise, we mimicked an economic ‘boom’ through a sudden, one‐time phosphorus addition into a fungus‐only compartment. This extra P was available to the fungus only, not the host root (Fig. 1d). These treatments were compared with the control treatment in which resource access remained constant (Fig. 1b).

To be able to follow this dynamic over time, we sequentially harvested replicates over a 3‐wk period. In each replicate, we quantified when the QD‐apatite was transferred by the fungus, from which compartment (Fig. 1), and how much was transferred. Using fungal biomass as a proxy for host carbon allocation (Olsson, 2002; Hammer *et al*., 2011b; Fortuna *et al*., 2012; Engelmoer *et al*., 2014; Whiteside *et al*., 2019; van’t Padje *et al*., 2020), we then calculated how the exchange rate changed as conditions became more (or less) favourable for the fungus.

Ultimately, our aim was to determine whether a resource boom was associated with a less favourable exchange rate for the fungus. This would be expected if the host plant was paying less C per unit of P received (i.e. a decrease in C : P ratio). Alternatively, if the fungus was successful at mediating the exchange rate in its favour – potentially via retention rather than immediate trading of the resource – we should see no change or a more favourable exchange rate associated with the addition of a resource pulse. We therefore calculated how the fungal network gained biomass in relation to the amount of nutrients transferred from the manipulated and stable fungus‐only compartments.

## Materials and methods

### Experimental design

We used a three‐compartment *in vitro* root mycorrhizal system in which the host root was grown in the core root compartment colonised by a fungal network that extended across two separate fungus‐only compartments (Fig. 1a). Resource increases and decreases were initiated in a single ‘manipulated’ fungus‐only compartment, while the other half of the fungal network grew under control conditions in the ‘stable’ fungus‐only compartment (Fig. 1c,d). P was supplied to each of the three compartments as QD‐apatite in different colours: red QD‐apatite to the core root compartment, yellow QD‐apatite to the manipulated fungal compartment and cyan QD‐apatite to the stable fungal compartment (Fig. 1a). In all replicates, the fungus was physically connected across the compartments. However, two plastic lips and the higher plastic barrier between the two fungal compartments restricted any nonfungal network movement of added nutrients (Whiteside *et al*., 2019). This ensured that all movement of tagged P was restricted to fungal network transport.

### Cultures and growing conditions

We grew *in vitro* Ri T‐DNA transformed carrot root (*Daucus carota*), inoculated with a single strain of arbuscular mycorrhizal fungus *Rhizophagus irregularis* (strain A5 Sanders Laboratory) in a three‐compartment 9‐cm Petri dish (Fig. 1a). Because only a single strain was used, this is considered ‘monopolistic competition’ (Hammerstein & Noë, 2016). We filled the core root compartment with Modified Strullu–Romand (MSR) medium (Fortin *et al*., 2002; Declerck *et al*., 2005; Kiers *et al*., 2011; Engelmoer *et al*., 2014), and the two fungus‐only compartments with a modified version of MSR medium that contained no additional P other than present in the solidifying agent (Phytagel, Sigma–Aldrich, St. Louis, MO, USA). For each treatment, we transferred 2 cm of a 3‐wk‐old branching root segment to the core root compartment. Directly after transfer, we inoculated the root by adding a 1 × 1 cm^2^ square of MRS medium with *c*. 400 spores and hyphae from a 3‐month‐old root organ culture (Engelmoer *et al*., 2014). Following colonisation of the host root, the fungal network, but not the roots, crossed over the plastic lips into the fungal compartments (Fig. 1a). We stored plates in the dark at 25°C, tilted at a 45° angle with the fungal compartments elevated, as an additional measure to prevent the roots from crossing to fungal compartments.

### QD‐apatite

To quantify the movement of P from three separate compartments and into the host, we tagged hydroxyapatite, a natural occurring rock phosphate (Ness & Vlek, 2000; Pel *et al*., 2018), with fluorescent nanoparticles (Whiteside *et al*., 2019). Apatite is difficult for plant roots to dissolve directly, however arbuscular mycorrhizal fungi can help break down apatite (Reynolds *et al*., 2006; Pel *et al*., 2018). Past work has shown that arbuscular mycorrhizal fungi can increase the dissolution and uptake of apatite, even under sterile conditions when no other microbes are present (Reynolds *et al*., 2006; Pel *et al*., 2018; van’t Padje *et al*., 2020). We constructed three colours of QD‐apatite (cyan (490 nm), yellow (576 nm) and red (666 nm)) that were equal in size and mass. We synthesised the QD‐apatite solution by adding 150 mg l^−1^ of carboxyl CdSeS core nanocrystals (Crystalplex, Pittsburg, PA, USA) into 1 L 50% modified simulated body fluid (MSBF) : 50% simulated body fluid (SBF) solution (11.9919 g NaCl; 1.96577 g NaHCO_3_; 0.447 g KCl; 0.4574 g MgCl_2_6H_2_O; 0.261 g K_2_HPO_4_; 0.4162 g CaCl_2_; 0.1062 g Na_2_SO_4_) (Tang *et al*., 2010; Kawashita *et al*., 2012; Whiteside *et al*., 2019). The carboxyl terminals of the QDs served as an anionic binding site to coat the QDs in apatite via the MSBF–SBF solution. To conjugate crystals, we performed a two‐phase reaction. In the first reaction, we exposed the solutions to 37°C for 24 h to conjugate the reagents to small crystals of *c*. 8 nm diameter. After this initial reaction, we placed the solutions at room temperature on a shaker for 60 h (100 oscillations per min). We then exposed the solutions to another 48 h to 37°C to initiate the second to conjugated bigger crystals of *c*. 200 nm, closely mimicking natural apatite (Sun *et al*., 2014). To remove unbound reagents, we washed the solutions twice by replacing 80% of the supernatant with Nanopure H_2_O, we shook the solutions by hand to re‐precipitate in between. We characterised the surface structure of the crystalised QD‐apatite using X‐ray photoelectron spectroscopy and determined that each nmol QD‐apatite contained *c*. 700 nmol of P (nmol P : QD = 708 : 1, Whiteside *et al*., 2019). We brought the solutions to a concentration of 1.39 mM P by diluting the solutions with Nanopure H_2_O. We autoclaved the solutions and stored them in the dark at 4°C. All technical controls (toxicity controls, colour controls, verification of fluorescing compounds in fungal and plant tissue, diffusion controls and unconjugated controls in which QDs lacked apatite and were not taken up) and method development are all described in detail in Whiteside *et al*. (2019).

At 5 wk after fungal inoculation, to each biological replicate (25 replicates per treatment per timepoint) we added three QD‐apatite solutions. In the stable fungal compartment, we added 0.33 ml cyan QD‐apatite. In the manipulated fungal compartment, we added 0.33 ml yellow QD‐apatite solution. In the root core compartment, we added 0.165 ml of the red QD‐apatite. At 1 wk after QD‐apatite addition, we initiated the resource increase and decrease treatments. For a resource increase, we doubled the amount of P available from that compartment by introducing an additional 0.33 ml yellow QD‐apatite solution to the manipulated fungal compartment. For a resource decrease, we physically severed and removed all hyphae in a 2‐mm strip of the MSR medium that was crossing the barrier between the manipulated compartment and the core root compartment and the hyphae were crossing the barriers to the manipulated compartment. The control plates experienced neither hyphal severing nor addition of extra QD‐apatite.

### Harvest

We destructively harvested 20 plates from each treatment at days 7, 14 and 21 after QD addition or severing events, and discarded contaminated plates (control: 26 out of 75, increase: 27 out of 75, decrease: 28 out of 75). We removed roots from the core root compartment with tweezers and placed them in paper bags to be dried for 48 h at 50°C. We placed the MSR medium, containing the extraradical mycelia from each compartment in 50 ml centrifuge tubes (Greiner Bio‐One International GmbH, Kremsmünster, Austria) and stored them for at least 24 h at −80°C to stop all metabolic reactions. We recorded dry weight (DW) of the roots and collected subsamples of roots (*c*. 7 mg) for epifluorescence analysis and for DNA extraction (*c*. 20 mg). To extract the extraradical fungal biomass, we dissolved the MSR medium from each compartment by adding 25 ml 10 mM sodium citrate solution. We then incubated the tubes in a water bath for 2 h at 65°C. We vacuum filtered the dissolved MSR medium over a 47 mm cellulose nitrate Whatman membrane filter (0.45 µm). As previously described, we then carefully removed the membrane filter from the vacuum filter and placed it on aluminium foil. We then slid a metal spatula systematically along the membrane filter to collect all extraradical fungal hyphae (Engelmoer & Kiers, 2014). We freeze dried the extraradical mycelia for 24 h (Engelmoer *et al*., 2014). We pulverised both root and extraradical mycelia samples using glass beads and a bead beater for 40 s on the highest speed (Thermo Savant FastPrep Fp120 Cell homogeniser).

### DNA isolation and real time qPCR

To determine extraradical fungal growth and intraradical fungal abundance, we isolated DNA from the roots and the extraradical mycelia using a DNeasy Plant Mini Kit (Qiagen, Hombrechtikon, Switzerland). We followed the manufacturer’s extraction protocol, with the exception that, after the lysis step, we added 10 µl of an internal standard, a plasmid of the cassava mosaic virus to control for DNA extraction efficiency (Whiteside *et al*., 2019). After DNA extraction, we quantified fungal abundance by measuring the copy numbers of the mtDNA SSU locus with *Taq*Man probe‐based qPCR using a CFX96 PRC detection system (Bio‐Rad, Hercules, CA, USA). We diluted root samples 100‐fold, and prepared all DNA samples for qPCR. We exported the resulting Cq values at a baseline threshold of 500 relative fluorescence units, and converted Cq values to copy numbers as described by Kiers *et al*. (2011). We then converted the mtDNA copy numbers to biomass (Voříšková *et al*., 2017) with a calibration curve described by Whiteside *et al*. (2019).

### Fluorescence analysis

We next quantified QD‐apatite fluorescence in the roots (Whiteside *et al*., 2019). We prepared the ground roots by adding 150 µl 10 mM borate buffer per mg of root. From each root sample, we took five replicates of 150 µl and pipetted them in a 96‐well plate with a glass bottom (Eppendorf AG, Hamburg, Germany). To circumvent edge effects, we left the outermost wells empty. We measured the emission spectra using a standard 96‐well epifluorescence microplate reader (Syngery• Mx monochromator‐based multimode microplate reader; BioTek, Winooski, VT, USA). We measured the emission at 325 nm excitation, ranging from 450 to 800 nm, with interval steps of 2 nm.

We translated the emission spectra to QD‐apatite concentrations using emission finger printing. This technique allowed us to separate the emission curves from the three differently coloured QDs even if these curves were overlapping. We used a custom script in Matlab Code (MathWorks, Natick, MA, USA) to detect low levels of QDs (> 0.000 001 nmol quantum dot per mg of plant tissue). For specific details see Whiteside *et al*. (2019). We converted fluorescence intensities to specific QD‐apatite transfer rates using a calibration gradient of QD‐apatite for each colour, composing of seven concentrations: 13.1, 9.83, 7.37, 5.53, 4.15, 3.11 and 2.33 mM.

### Statistical analysis

We performed all statistical analyses in R v.3.3.1. For each response variable, we checked the residuals for normality with a Shapiro–Wilk test. We checked the distribution of the residuals with QQ plots and analysed the homogeneity of variance across groups using Levene's test. We separately analysed root growth, the uptake of three colours of QD‐apatite in host roots, the extraradical fungal abundance, the logarithm of the intraradical fungal abundance and the total amount of QD‐apatite (sum of the three colours) with a linear model using treatment (resource increase, resource decrease or control), the time of harvest (ordered categorical variable), and the treatment × harvest interaction as explanatory variables.

Finally, we analysed resource exchange rate as the logarithm of C allocation to the fungus to P transfer to the root. We measured C allocation as the sum of fungal copy numbers in the two fungal compartments and P transfer as the sum of QDs from those compartments per host root. To analyse how the exchange rate changed over time, we analysed each treatment with a linear model with an explanatory ordered categorical variable the days after the event. To look for absolute differences between the two treatments, we tested the differences between the resource exchange rates at specific time points with a Wilcoxon rank sum test because the data were not normally distributed.

## Results

### Transfer of P across fungal network

We first determined total amount of QD‐apatite transfer to host roots from the fungal network exposed to the resource treatments. By quantifying and summing the fluorescence of all three colours, we found that total QD‐apatite transferred to the host roots increased over time, but with no significant difference across treatments (Supporting Information Fig. S1). This gradual increase over time is an indication that the QD‐apatite acts similarly to nontagged apatite transferred from mycorrhizal fungi (Pel *et al*., 2018). Specifically, the fungus transferred an average of 0.143 ± 0.006 nmol QD‐apatite per total root, or 0.002 ± 0.0001 nmol per mg of root over the 21 d after treatments were initiated. This amount was similar that found in previous studies with QD‐apatite (Whiteside *et al*., 2019; van’t Padje *et al*., 2020). Similarly, total root biomass was not significantly affected by nutrient treatments to fungal network (Fig. S2). However, roots became larger over time, independent of the ‘crash’ or ‘boom’ resource treatment. This further confirms that QD‐apatite acts similarly to other P resources, and that the host root continued to receive sufficient P to sustain its growth, regardless of the resource treatment to the fungal network (Fig. S1).

Because we labelled the three apatite pools with QDs fluorescing with different colours (Fig. 1a), we could determine from which compartment the QD‐apatite was transferred (Fig. S3). Here, we found a strong treatment effect: severing the fungal network in the resource decrease treatment was associated with a significantly greater QD‐apatite contribution from the core root compartment compared to the core treatment of the control treatment and resource increase treatment, but not different when comparing the control and the resource increase treatment (Fig. 1e). We also did not find evidence that exposing the fungal network to a pulse of QD‐apatite in the boom treatment increased QD transfer from that pool to the root (Fig. S3). Despite a doubling of resource availability to the fungus, we did not observe a significant increase in transfer to the host (Fig. S3).

### Fungal growth patterns in response to pulses and restrictions of nutrients

**Fig. 2:**
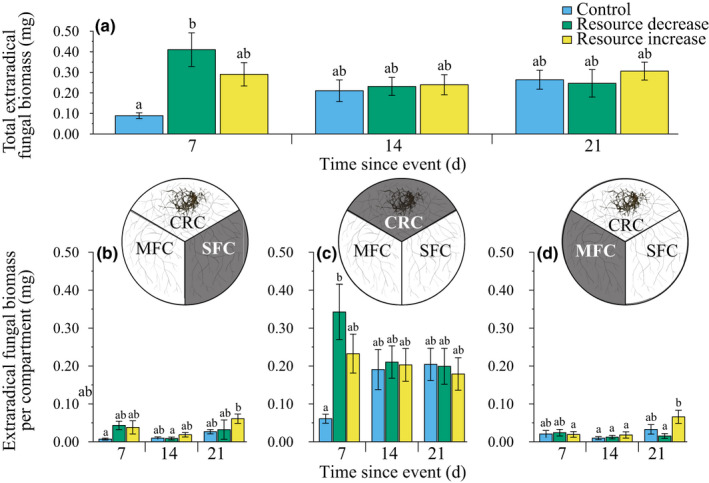
Extraradical fungal biomass (*Rhizophagus irregularis*) over time. (a) Total extraradical fungal biomass was significantly influenced by treatment and the interaction between treatment and time (two‐way ANOVA for treatment: *F*_2,132_ = 3.091, *P* = 0.049; time: *F*_2,132_ = 0.949, *P* = 0.390; interaction: *F*_4,132_ = 2.898, *P* = 0.024). Total extraradical fungal biomass steadily increased over time in the control treatment (blue). In the resource decrease treatment (green) the total fungal biomass was highest 7 d after the event, then decreased. In the resource increase treatment (yellow) the fungal biomass did not change over time. (b) In the stable fungal compartment, fungal biomass was significantly influenced by treatment and time effect (two‐way ANOVA for treatment: *F*_2,132_ = 3.655, *P* = 0.029; time: *F*_2,132_ = 5.071, *P* = 0.008; interaction: *F*_4,132_ = 1.076, *P* = 0.371). (c) In the core root compartment (CRC) the fungal biomass was a four‐fold higher than in the two fungal compartments. The fungal biomass in the CRC was significantly influenced by the interaction between treatment and time (two‐way ANOVA for treatment: *F*_2,132_ = 2.606, *P* = 0.078, time: *F*_2,132_ = 0.071, *P* = 0.931; interaction: *F*_4,132_ = 2.770, *P* = 0.030). (d) In the manipulated fungal compartment, the fungal biomass was not significantly influenced treatment, only by time (two‐way ANOVA for treatment: *F*_2,132_ = 2.119, *P* = 0.124; time: *F*_2,132_ = 5.000, *P* = 0.008; interaction: *F*_4,132_ = 2.064, *P* = 0.089). *n*_control,7_ = 14, *n*_control,14_ = 14, *n*_control,21_ = 21, *n*_decrease,7_ = 16, *n*_decrease,14_ = 18, *n*_decrease,21_ = 13, *n*_increase,7_ = 16, *n*_increase,14_ = 17, *n*_increase,21_ = 14. Mean ± SE. CRC, Core root compartment; MFC = manipulated fungal compartment; SFC, stable fungal compartment. Bars with the same letter do not have a significantly different mean extraradical fungal biomass based on post hoc test.

We found a significant effect of treatment on the extraradical fungal biomass of the fungal network (sum of all compartments). Specifically, severing the fungal network stimulated hyphal growth at day 7, followed by a subsequent decrease and stabilisation of biomass at a lower level compared with other treatments (Fig. 2a). No such stimulation was found in overall biomass measurements of the fungal network exposed to the resource increase or control treatments (Fig. 2a).

Because the fungal network was divided into sections exposed both directly (manipulated compartment) and indirectly (stable compartment) to the resource treatments, we could then compare how different parts of the same network grew in response to an influx and restriction of nutrients. First, we quantified the fungal network biomass in the stable compartment, where we found a significant treatment effect. Resource addition was characterised by a burst of extraradical hyphae growth, which was most pronounced 21 d after nutrient addition, whereas the resource decrease treatment showed erratic increases and decreases of biomass. In the control treatment, we documented a steady increase in fungal biomass over time, as expected (Fig. 2b). We did not find a treatment nor timing effect on the extraradical fungal biomass in the core root compartment (Fig. 2c), nor in the manipulated fungal compartment (Fig. 2d). The only significant trend in the manipulated fungal compartment was again a bust of extraradical fungal biomass 21 d after the nutrient addition (Fig. 2d).

**Fig. 3:**
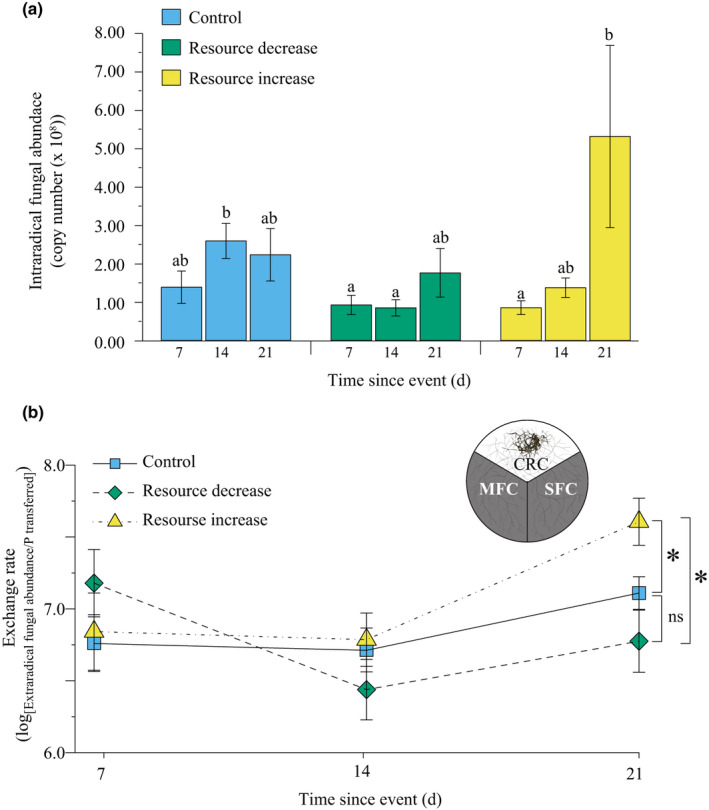
Intraradical fungal abundance (*Rhizophagus irregularis*) and exchange rate (extraradical fungal abundance/P transferred). (a) Intraradical fungal abundance was significantly influenced by treatment and time (two‐way ANOVA for treatment: *F*_2,130_ = 4.953, *P* = 0.008; time: *F*_2,130_ = 6.804, *P* = 0.002; interaction: *F*_4,130_ = 1.408, *P* = 0.235). In the control treatment (blue) the fungal abundance steadily increased over time. The fungal abundance was lowest in the resource decrease treatment (green). In the resource increase treatment (yellow) the fungal abundance peaked 21 time after the event. *n*_control,7_ = 7, *n*_control,14_ = 14, *n*_control,21_ = 22, *n*_decrease,7_ = 14, *n*_decrease,14_ = 20, *n*_decrease,21_ = 13, *n*_increase,7_ = 19, *n*_increase,14_ = 16, *n*_increase,21_ = 14; means ± SE. Bars with the same letters do not have a significantly different mean, based on post hoc test. (b) We found a significant time effect on the exchange rate, defined as the amount of carbon (C) received by the fungus over the amount of phosphorus (P) transferred to the host root, expressed as the sum of the fungal abundance in the two fungal compartments over the sum of P transferred from the two fungal compartments, in the resource increase treatment (yellow triangles) (one‐way ANOVA: *F*_2,44_ = 4.341, *P* = 0.019), but not in the other two treatments (one‐way ANOVA: control (blue squares): *F*_2,46_ = 2.308, *P* = 0.111; resource decrease (green diamonds): *F*_2,42_ = 2.953, *P* = 0.063). At day 21, we found a significantly higher exchange rate for the resource increase treatment compared with the control (Wilcoxon rank sum test *P* = 0.002) and the resource decrease treatment (Wilcoxon rank sum test *P* = 0.003), indicated by an asterisk. *n*_control,7_ = 14, *n*_control,14_ = 14, *n*_control,21_ = 21, *n*_decrease,7_ = 14, *n*_decrease,14_ = 18, *n*_decrease,21_ = 13, *n*_increase,7_ = 16, *n*_increase,14_ = 17, *n*_increase,21_ = 14, means ± SE. CRC, Core root compartment; MFC, manipulated fungal compartment; SFC, stable fungal compartment.

A symbiotic fungus can change its trading strategy by expanding the size of its fungal network, or by increasing colonisation inside the root where the trading takes place. We therefore measured fungal abundance inside host roots (i.e. intraradical colonisation) via quantitative PCR (Kiers *et al*., 2011; Voříšková *et al*., 2017). We found that the intraradical fungal abundance was significantly influenced by treatment and time. Intraradical fungal abundance increased over time in all treatments, with the highest increase in the resource increase treatment 21 d after nutrient addition. In comparison, we found the lowest intraradical fungal abundance in the resource decrease treatment (Fig. 3a). This means that severing the mycelium in the resource decrease treatment was costly for the fungus, with 21% lower intraradical colonisation compared with the control treatment, and a 67% lower colonisation compared with the resource increase treatment.

### Changes in exchange rate

We found that the exchange rate was significantly different among harvest dates in fungal networks exposed to resource increase in the boom treatment, with an increase in C : P ratio over time (Fig. 3b). By the termination of the experiment (day 21), the fungal networks exposed to a resource increase had a significantly higher exchange rate as measured by total fungal biomass per unit of P transferred across the two fungal compartments, compared with the control and the resource decrease treatments. By contrast, there was no increase or decrease in exchange rate in the control or resource decrease treatment (Fig. 3b).

## Discussion

In the resource decrease treatment, we effectively reduced access of the fungal network to P by *c*. 40%. The fungus responded in two ways. First, severing the fungal network appeared to stimulate fungal growth, especially at day 7. This growth response mirrors mechanisms of regrowth documented in plants after being pruned (Prusinkiewicz *et al*., 2009), and growth in fungal gardens after pruning by leaf cutter ants (Bass & Cherrett, 1994, 1996), suggesting a process by which the fungus reallocated reserves from other parts of the fungal network. This was supported by evidence of intraradical colonisation, which was lower in the severing treatment, especially at day 14, possibly as a result of resources being reallocated to growing tips (Fig. 3a). Such flexibility in biomass allocation is an important adaptation in fungal networks because extending hyphae are vulnerable to disturbances, consumption by animals or other physical damage (Moore *et al*., 1985; Jasper *et al*., 1989; Klironomos & Kendrick, 1996). Second, we found that the fungus responded to resource restriction by accessing an alternative P pool closer to the host root, changing where across the fungal network QD‐apatite was transferred. This resulted in more resource transfer from the core root compartment (Fig. 1e). This is the first evidence to suggest a compensation mechanism by the fungus, whereby when one source of QD‐apatite was lost, transfer is augmented from another source across the fungal network. In this case, the augmented source was closer to the root, where the fungal network is likely to be more dense, compared with further away from the root (Thonar *et al*., 2011). This also suggested that the fungal network is able to cope with major changes in external resource environment, including both the physical severing of the fungal network and doubling of the available QD‐apatite, and still maintain a steady supply of nutrients to hosts.

An alternative explanation for the increase in resources from the core root compartment (Fig. 1e) is that the host root – which also has direct access to the QD‐apatite in the core root compartment – increased its own uptake when the extended fungal network failed to deliver sufficient P. While we could not rule out this possibility, this was unlikely. First, we utilised apatite, a form of rock P that is difficult for plant roots to obtain directly, but that arbuscular mycorrhizal fungi can help break down (Reynolds *et al*., 2006; Pel *et al*., 2018). Our aim was to use a form of phosphorus that was difficult for the host to take up directly (Reynolds *et al*., 2006; Pel *et al*., 2018), essentially increasing the ‘bargaining’ position of the fungi (Noë & Kiers, 2018). Past work on split‐root systems has shown that root systems colonised with arbuscular mycorrhizal fungi are better able to take up of apatite compared with noncolonised roots (Whiteside *et al*., 2019). Once inside the root, the QD‐apatite is transported to the growing leaves, suggesting that QD‐tagged nutrients are transported in a similar manner to other P sources (Whiteside *et al*., 2009). While the exact apatite uptake mechanism for arbuscular mycorrhizal fungi is still unknown, fungi generally use endocytic pathways to take up large particles, with invaginating cells reaching diameters of 100 nm (Fischer‐Parton *et al*., 2000; Read & Kalkman, 2003; Epp *et al*., 2013; Lu *et al*., 2016). The ability of the fungus to take up apatite was supported with bright‐field imaging videos of nutrient flows that showed vacuoles inside hyphae when the fungus was given access to QD‐tagged apatite and the absence of large vacuoles when QD‐tagged apatite is absent (van’t Padje *et al*., 2020). Second, while no studies directly investigated whether *R. irregularis* can repress the ‘direct uptake’ pathway of P by *D. carota* roots, the fungus *R. irregularis* can repress direct P uptake in roots of *Medicago truncatula* (Watts‐Williams *et al*., 2015) and there is evidence that colonisation by arbuscular mycorrhizal fungi can suppress the direct P uptake in several other roots (Smith *et al*., 2004; Javot *et al*., 2007; Grønlund *et al*., 2013; Watts‐Williams *et al*., 2015), such that highly colonised mycorrhizal roots are unlikely to rely solely on the direct uptake pathway. We confirmed this increased host reliance on the fungus in the core compartment by measurement of intraradical colonisation that, although low compared with other treatments, increased rather than decreased over time in the resource decrease treatment (Fig. 3a). Third, past work has shown that plant hosts can control of levels of intraradical colonisation depending on levels of available nutrients. For example, plant roots suppress the formation of intraradical fungal structures if nutrient levels are high (Vierheilig *et al*., 2000; Catford *et al*., 2003; Gu *et al*., 2011; Foo *et al*., 2014), and can actively regulate the amount of C located to arbuscular mycorrhizal fungi (Konvalinková *et al*., 2017). If the host was responsible for all the QD‐apatite uptake (vs QD‐apatite transfer from the fungal network), this would have been associated with less, rather than more, reliance on the fungus and a decrease in intraradical colonisation over time.

We next studied resource increase by exposing part of the fungal network to a pulse of QD‐apatite. Rather than an influx of QD‐apatite driving a decrease in the exchange rate (less C received per unit of P), we found that the fungus benefited from the resource pulse (Fig. 3b). These data suggested that the fungus was able to capitalise on the nutrient addition, perhaps by controlling the transfer of the phosphorus to the host over time (Fig. 3b). Under this scenario, the fungus would store the surplus nutrient – rather than immediately trading it – until plant demand, and thus value to the root, increased. This hypothesis was supported by several lines of evidence. First, despite doubling the amount of QD‐apatite to the fungal network, we did not observe any statistically significant increase in overall amount of QD‐apatite transferred to the host root in the boom treatment (Figs S1, S3). This is likely to be a reflection of the fungus retaining the QD‐apatite in the fungal network over the time frame of our experiment. It has been hypothesised that such retention, as documented in other studies, is an effective strategy by which nutrients are stored until they become scarce again, allowing the fungus to potentially gain a better exchange rate over time (Hammer *et al*., 2011b; Kiers *et al*., 2011; Whiteside *et al*., 2019; van’t Padje *et al*., 2020). Second, we found an increase in total fungal abundance over time in both the stable and manipulated fungal compartments in the resource increase treatment (Fig. 2b,d). Such an increase in biomass increase is not expected if the nutrients from the P pulse remained immobilised, for example in the medium. This biomass increase was not found in the resource decrease or control treatments. This suggested that fungal networks exposed to the resource boom were gaining a C benefit that is realised over time, rather than a benefit realised immediately. This suggestion is supported by data on intraradical colonisation (Fig. 3a), in which the roots given the resource increase treatment showed the highest densities of intraradical fungal abundance compared with the other treatments, but only at the 21 d harvest. Higher densities of intraradical colonisation are necessary for higher exchange of P to C resources, and indicative of fungal growth and benefit (Grace *et al*., 2009; Campos *et al*., 2018). Lastly, if the nutrients were stored until their value increased, we should also be able to see if this was reflected in a changing exchange rate over time. We found that when exposed to a resource increase, the exchange rate increased over time, with a higher C : P ratio received for the fungus, a trend not found in the other treatments (Fig. 3b). At the 21 d harvest, the fungus exposed to the resource increase treatment had gained significantly more biomass per unit QD‐apatite compared with the control and resource decrease treatment samples. Given that this trend was only evident 21 d after the QD‐apatite pulse, it highlights the importance of studying exchange rates over multiple harvests, rather than at only one time point.

While these data are in line with the idea that the fungus should ultimately benefit from an addition of nutrients accessible only to the fungus, there is an open question of how to accurately measure host allocation to the fungal network. Because arbuscular mycorrhizal fungi are obligate biotrophs, all C gained by the fungus to form and maintain fungal networks is derived from the host (Jiang *et al*., 2017; Luginbuehl *et al*., 2017). We used biomass as a proxy for host allocation (Olsson, 2002; Hammer *et al*., 2011b; Fortuna *et al*., 2012; Engelmoer *et al*., 2014; Whiteside *et al*., 2019; van’t Padje *et al*., 2020). This approach did not take into account potential metabolic differences in the fungi among treatments. However, loss of carbon via respiration in arbuscular fungal networks is orders of magnitude lower than the accumulation of C biomass in the network. This has been estimated at *c*. 0.2 μg C g^−1^ d^−1^ (Heinemeyer *et al*., 2006). It is hypothesised that arbuscular mycorrhizal fungi have low carbon loss because their fine hyphal networks have relatively high carbon use efficiency (CUE), also reflective of high accumulation of lipids in their mycelia (Konvalinková *et al*., 2017). Ideally, C allocation should be calculated based on a similar method of tagging C with QDs, as we have done for P. This would also allow us to visually confirm where and when the host transferred C to the fungal network. However, it is also unknown if QD‐tagged hexose could ever serve as a relevant C allocation measurement. Tagging C with QDs remains methodologically challenging, and the product may be toxic (Rispail *et al*., 2014).

An important next step is to scale up our findings to whole plants. While root organ cultures are an established model system for metabolism and transport processes in the arbuscular mycorrhizal symbiosis (Bago *et al*., 2000; Fortin *et al*., 2002; Olsson, 2002; Declerck *et al*., 2005), we do not know how the lack of a direct shoot sink for P influences our results. Past work has demonstrated that root organ cultures possess similar nutrient and resource transfer and metabolic characteristics as whole‐plant systems (Olsson, 2002; Bago *et al*., 2003; Pfeffer *et al*., 2004; Govindarajulu *et al*., 2005; Olsson & Johnson, 2005; Olsson *et al*., 2005; Bücking & Shachar‐Hill, 2005; Tian *et al*., 2010; Hammer *et al*., 2011a), but these dynamics need to be tested more extensively with QD‐apatite in whole plants. We have shown previously that QD‐apatite can be taken up by whole *Medicago truncatula* plants and translocated and retained in the leaves and shoots, as expected under natural conditions (Whiteside *et al*., 2019). We have also confirmed that inoculation with arbuscular mycorrhizal fungi facilitated the uptake of QD‐apatite in plants compared with nonmycorrhizal controls. However, additional tests are now needed in which exchange rates of fungal networks are monitored in whole plants, with natural source‐sink dynamics, over time. One idea is to further develop an *in vitro* autotrophic whole‐plant systems in which the mycorrhizal root and mycelium develop in a 2D agar system (Voets *et al*., 2005). This would allow the high precision monitoring and quantification of nutrient transfer needed to test biological market theory, combined with more natural source‐sink dynamics (Gyuricza *et al*., 2010).

More generally, an open question is how these nutrient transfer dynamics change when more additional partners are introduced into the system. Using the QD‐tagging approach of three colours, we were able to document how trading strategies of a single laboratory‐cultured fungus in monopolistic competition varied spatially under resource perturbations. However, competition is another key aspect driving trade dynamics (Jones *et al*., 2012; Chamberlain *et al*., 2014). Under our experimental conditions, the fungal network had a monopoly on P resources: there was no competition from other fungi. As a result, there were no forces preventing the fungus from retaining, and thus driving up, the value of the QD‐apatite resource (Fig. 3b). In scaling up to more complex communities with multiple traders (e.g. Lekberg & Waller, 2016), there is the potential for underbidding by competitors that is predicted to drive down the price of P (Wyatt *et al*., 2014; Noë & Kiers, 2018). Our results suggested that fungal networks may act as a buffer for plants against extreme changes in resource environment. However, when harnessing root microbiomes for use in sustainable agriculture, multiple competing fungal partners will be present (Toju *et al*., 2018). While we have shown that the host received a consistent P supply – regardless of external nutrient perturbations to the fungal network – future work is needed to manipulate fungal diversity, and thus competitive dynamics, to determine where and how fungal trade strategies change across the network in the presence of other traders.

## Author contributions

AP designed and ran experiments. She performed statistical analyses, generated figures and led manuscript writing. GDAW was involved in the experimental design, statistical design and writing. ETK was involved in the experimental design, and manuscript writing.

## Supporting information


**Fig. S1** Total QD‐apatite per host root per treatment over time.
**Fig. S2** Dry root biomass treatment over time.
**Fig. S3** QD‐apatite per host root per treatment per compartment.
Please note: Wiley Blackwell are not responsible for the content or functionality of any Supporting Information supplied by the authors. Any queries (other than missing material) should be directed to the *New Phytologist* Central Office.

## Data Availability

Upon publication, all scripts, analyses and data, will be available from: https://github.com/anoukvantpadje/Market_crash.
