# Supplementary material for: Mycorrhizal fungi control phosphorus value in trade symbiosis with host roots when exposed to abrupt ‘crashes’ and ‘booms’ of resource availability
Source: New Phytol. 2020 Nov 29;229(5):2933–44. doi: 10.1111/nph.17055 (PMC7898638; doi:10.1111/nph.17055)
Supplement: Supplementary file 1 — Fig. S1 Total QD‐apatite per host root per treatment over time. Fig. S2 Dry root biomass treatment over time. Fig. S3 QD‐apatite per host root per treatment per compartment. Please note: Wiley Blackwell are not responsible for the content or functionality of any Supporting Information supplied by the authors. Any queries (other than missing material) should be directed to the New Phytologist Central Office. [file NPH-229-2933-s001.pdf]

New Phytologist Supporting Information

Article title: Mycorrhizal fungi control value of phosphorus in trade symbiosis with host roots when exposed to abrupt 'crashes' and 'booms' of resource availability

Authors: Anouk van 't Padje, Gijsbert D.A. Werner and E. Toby Kiers

Article acceptance date: 20 October 2020

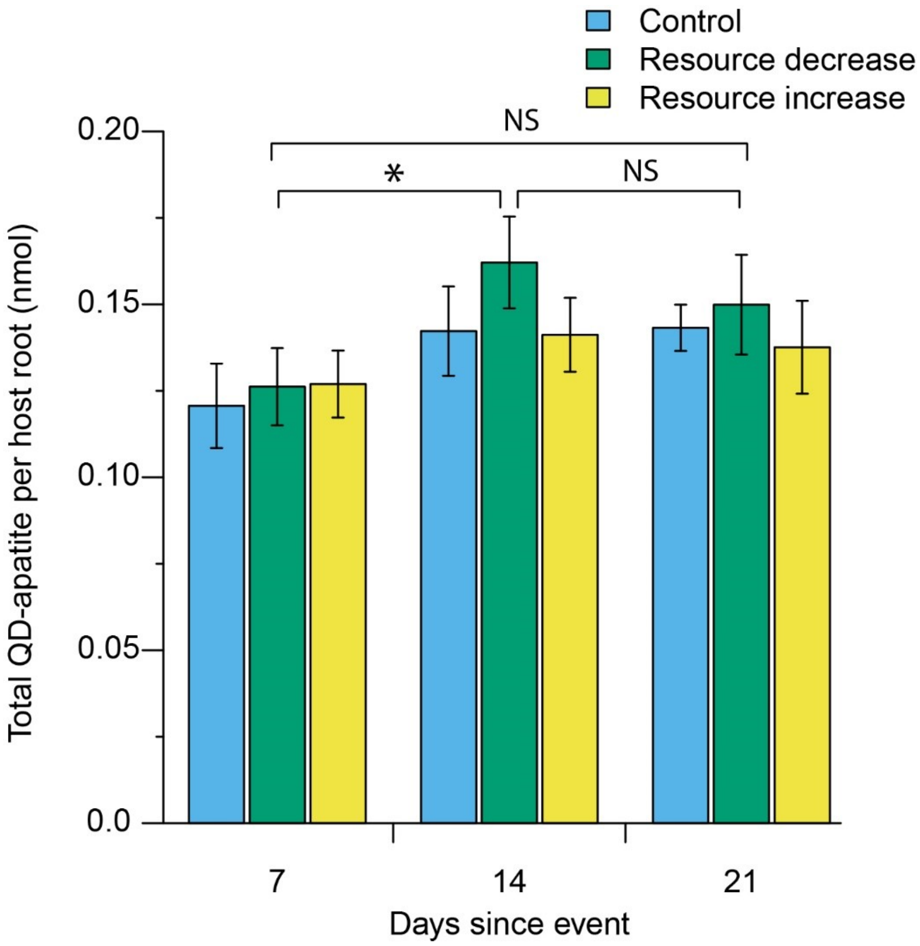

Fig. S1 Total Quantum-dot (QD)-apatite per host root (*Daucus carota*) per treatment over time. The total amount QD-apatite in the host roots was not significantly influenced by the treatments, only by time after the event (2-way ANOVA: treatment: $F_{2,132}$=0.914, p=0.403, time: $F_{2,132}$=3.553, p=0.031; interaction: $F_{4,132}$=0.287, p=0.887). $n_{control,7}$=14, $n_{control,14}$ =14, $n_{control,21}$=21, $n_{decrease,7}$=16, $n_{decrease,14}$=18, $n_{decrease,21}$=13, $n_{increase,7}$=16, $n_{increase,14}$=17, $n_{increase,21}$=14. Mean ±SEM. Significant effects of time on QD-apatite in host roots are indicated with an *, non-significant effects with NS.

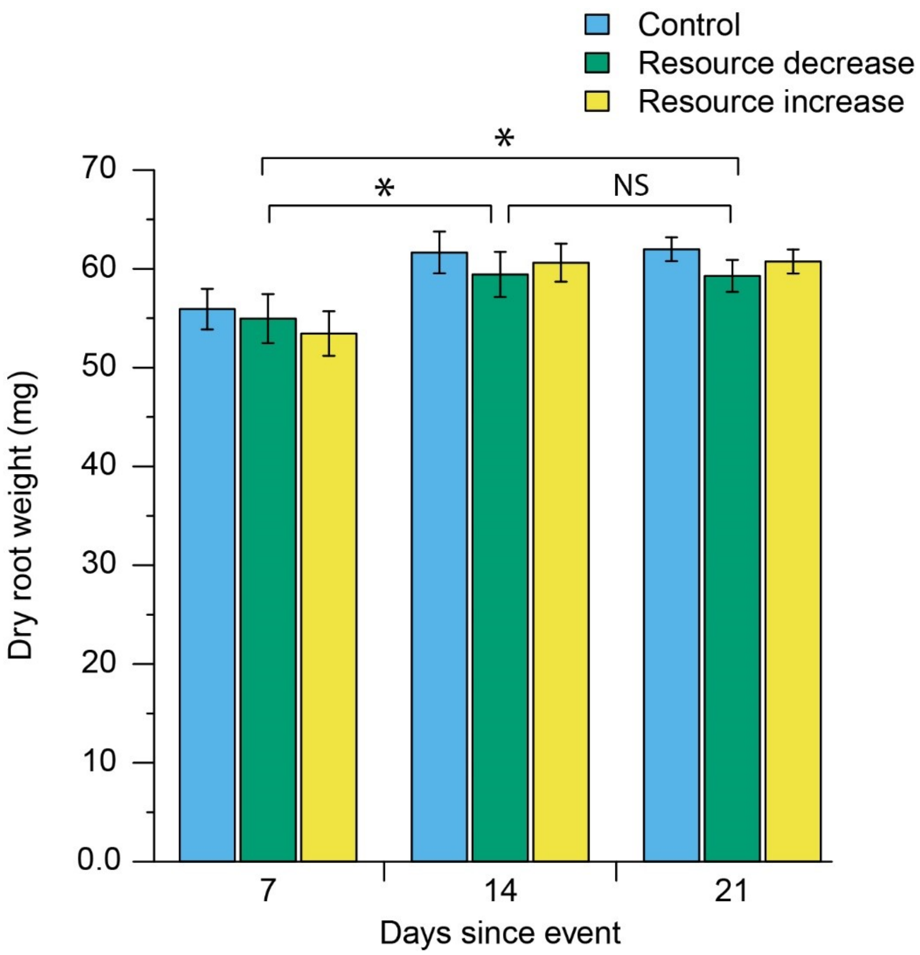

Fig. S2 Dry root biomass (*Daucus carota*) treatment over time. The roots grew significantly over time, but the biomass was not significantly influenced by the treatments (2-way ANOVA: treatment: $F_{2,132}$=0.879, p=0.418, time: $F_{2,132}$=8.086, p<0.001; interaction: $F_{4,132}$=0.173, p=0.952). $n_{control,7}$=14, $n_{control,14}$ =14, $n_{control,21}$=21, $n_{decrease,7}$=16, $n_{decrease,14}$=18, $n_{decrease,21}$=13, $n_{increase,7}$=16, $n_{increase,14}$=17, $n_{increase,21}$=14. Mean ±SEM. Significant effects of time on the dry root weight are indicated with an *, non-significant effects with NS.

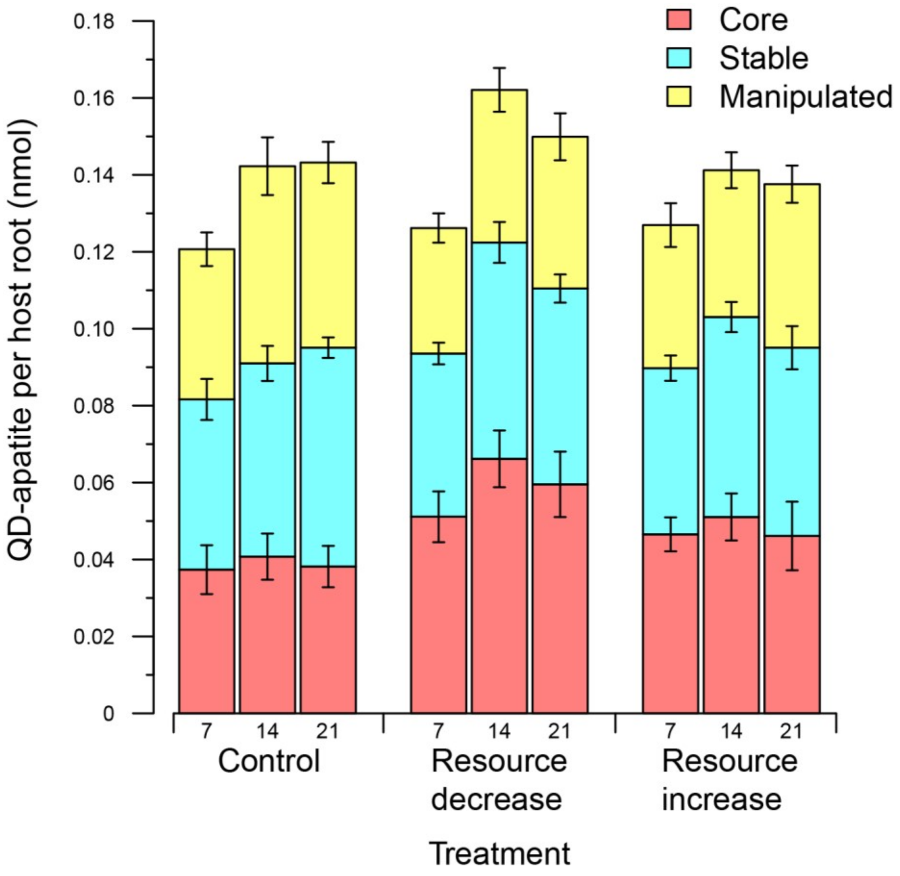

Fig. S3 Quantum-dot (QD)-apatite per host root (*Daucus carota*) per treatment per compartment. The origin of the QDs is indicated by the color or the stacked bars. The amount of QDs transferred from core compartment is significantly influenced by the treatment, with higher transfer in the resource decrease treatment (2-way ANOVA: treatment: $F_{2,132}$=6.955, p=0.001, time: $F_{2,132}$=1.035, p=0.358; interaction: $F_{4,132}$=0.248, p=0.911) (Fig. 2). The amount of QD-apatite transferred from the stable fungal compartment (cyan) or from manipulated fungal compartment (yellow) was not significantly influenced by the treatment (2-way ANOVA: stable compartment: treatment: $F_{2,132}$=0.358, p=0.700, time: $F_{2,132}$=4.998, p=0.008; interaction: $F_{4,132}$=0.676, p=0.610; manipulated compartment: treatment: $F_{2,132}$=2.125, p=0.124, time: $F_{2,132}$=1.436, p=0.242; interaction: $F_{4,132}$=0.268, p=0.898). $n_{control,7}$=14, $n_{control,14}$ =14, $n_{control,21}$=21, $n_{decrease,7}$=16, $n_{decrease,14}$=18, $n_{decrease,21}$=13, $n_{increase,7}$=16, $n_{increase,14}$=17, $n_{increase,21}$=14. Mean ±SEM.
